# A Theoretical Analysis on a Multi-Bed Pervaporation Membrane Reactor during Levulinic Acid Esterification Using the Computational Fluid Dynamic Method

**DOI:** 10.3390/membranes11080635

**Published:** 2021-08-17

**Authors:** Milad Ghahremani, Kamran Ghasemzadeh, Elham Jalilnejad, Adolfo Iulianelli

**Affiliations:** 1 Faculty of Chemical Engineering, Urmia University of Technology, Urmia 5756151818, Iran; milad.gharemani@uut.ac.ir (M.G.); e.jalilnejad@uut.ac.ir (E.J.); 2 Institute on Membrane Technology of the Italian National Research Council (CNR-ITM), Via P. Bucci cubo 17/C, 87036 Rende, CS, Italy

**Keywords:** pervaporation membrane reactor, modeling and simulation, esterification process, computational fluid dynamic (CFD) method

## Abstract

Pervaporation is a peculiar membrane separation process, which is considered for integration with a variety of reactions in promising new applications. Pervaporation membrane reactors have some specific uses in sustainable chemistry, such as the esterification processes. This theoretical study based on the computational fluid dynamics method aims to evaluate the performance of a multi-bed pervaporation membrane reactor (including poly (vinyl alcohol) membrane) to produce ethyl levulinate as a significant fuel additive, coming from the esterification of levulinic acid. For comparison, an equivalent multi-bed traditional reactor is also studied at the same operating conditions of the aforementioned pervaporation membrane reactor. A computational fluid dynamics model was developed and validated by experimental literature data. The effects of reaction temperature, catalyst loading, feed molar ratio, and feed flow rate on the reactor’s performance in terms of levulinic acid conversion and water removal were hence studied. The simulations indicated that the multi-bed pervaporation membrane reactor results to be the best solution over the multi-bed traditional reactor, presenting the best simulation results at 343 K, 2 bar, catalyst loading 8.6 g, feed flow rate 7 mm^3^/s, and feed molar ratio 3 with levulinic acid conversion equal to 95.3% and 91.1% water removal.

## 1. Introduction

Global warming caused by the growing greenhouse gases generation has been currently recognized as a major environmental problem. The use of petroleum-derived fossil fuel and the concomitant vegetation areas reduction are the main causes of a rise in CO_2_ concentration in the atmosphere. Apart from the CO_2_ effect, an increase in other industrial byproducts such as chlorofluorocarbons, hydrocarbons, methane, nitrogen oxides (NOx), and sulfur oxides (SOx) also increase the greenhouse effect. From an environmental point of view, the main advantage of using biofuels is reducing the effect of greenhouse and acid rain. As a renewable fuel option, in the near future, the importance of using biodiesel and bio-ethanol and, in the medium future, the importance of using biomass-derived Fischer-Tropsch diesel and cellulosic biomass-based additives will increase. Biomass-based ethyl levulinate (EtLA) is a significant fuel additive. The use of EtLA as a bio-fuel provides higher engine efficiency and lower CO and NOx emissions in addition to providing long operating life. Since diesel engines are responsible for high NOx and exhaust gas emissions, they have negative effects on the environment. To solve this issue, one of the most effective methods consists of the addition of oxygenates to fossil fuels, which are known as alkyl levulinate as a fuel oxygenate. EtLA attracted special attention due to its properties of high ignition temperature, 33% oxygen content, and high-efficiency clean combustion [1,2,3,4,5,6]. Levulinate esters are important chemical compounds used as fuel additives, solvents, and plasticizers. Ethyl levulinate can be used as a bio-additive in diesel fuel up to 5 wt%. The EtLA is synthesized by esterification of levulinic acid (LA) with ethanol (Et) in the presence of homogeneous or heterogeneous acid catalysts. Both reactants are produced from bio-based sources. The use of bio-based reactants makes the production of EtLA process green and environmentally friendly [7,8]. EtLA is usually synthesized by using acid catalysts such as H_2_SO_4_, HCl, and H_3_PO_4_. They are unrecyclable and corrosive. Therefore, in recent years, many research groups explored the potential of green catalysts use. Heterogeneous solid acid catalysts are a suitable alternative to overcome the drawbacks of homogeneous acid catalysts. Heterogeneous catalysts can be separated from the reaction mixtures easily and reused for repeated runs [8,9,10,11].

Nowadays, membrane processes belong to a large group of techniques for separating the components of liquid and gas mixtures, depending on the properties of the used membranes and the specific characteristics of the separation itself. Different membrane operations are currently adopted for separating products ranging in size from tens of µm to tenths of nm, and particular types of membranes allow for the separation of vapors, gases, ions, etc. (reverse osmosis, nanofiltration, and membrane distillation). Among them, membrane pervaporation (PV) is useful for separating liquid mixtures, including those forming azeotropes. It depends on the selective sorption and diffusion rate differences and not from the liquid-vapor equilibrium. Most of the polymeric membranes used currently in PV processes may be categorized as in the following:

- Inert membranes possessing hydrophilic nature (i.e., polyvinyl alcohol, cellulose acetate);
- Inert membranes possessing hydrophobic nature (i.e., polydimethylsiloxane);
- Hydrophilic ion exchange membranes.

Furthermore, membrane engineering deals also with the combination of two or more operation units in order to provide benefits such as the reduction in the cost of investment and the energy consumption. In this regard, pervaporation membrane reactors (PVMRs) may perform both the reaction and separation stages simultaneously. The application of PVMR enhances the conversion of reversible reactions as a result of the continuous removal from the reaction mixture of the byproduct with a membrane. Esterification of carboxylic acids and alcohols is a typical example of an equilibrium-limited reaction that produces ester and water as products. Limited by the equilibrium conversion, the aforementioned process generally shows low conversion, and an excess of reactants is required to enhance its value, determining an increase in the costs. Accordingly, removing reaction products from the reaction side, for instance, an ester or water, by pervaporation allows that, for the Le Chatelier principle, the reaction may be shifted toward the products. The esterification of LA with Et to produce EtLA and water is reported in the following [12,13,14,15,16,17,18,19,20]:

LA + Et ↔ EtLA + H_2_O (1)

Numerical models could be useful to avoid high experimental costs and to develop a better understanding of the effects of various parameters for the design and the fruitful application of PVMR in the levulinic acid esterification (LA-ESR) as well as for specific features and constraints such as the need of obtaining high conversion. To this purpose, the computational fluid dynamic (CFD) tool is a feasible method to simulate detailed liquid flow characteristics at any point of a membrane system. Indeed, the CFD approach can be used for the virtual prototyping of chemical reactors and separators. Since it is based on controlled volume methodology, the local variations of the fluid, thermal, and mass transport properties can be visualized in comparison to simple models and used to design a PVMR [21,22,23,24]. Nevertheless, to our best knowledge, there are not CFD-based studies about the application of PVMR for the LA-ESR. In this work, the LA-ESR reaction in a multi-bed PVMR (MB-PVMR) was theoretically analyzed, using the CFD method in order to evaluate the effects of the most significant operating parameters such as the reaction temperature, catalyst loading, feed molar ratio, and feed flow rate on the performance of the MB-PVMR in terms of LA conversion and water removal. Furthermore, the MB-PVMR performance was compared with those of a multi-bed traditional reactor (MB-TR) and discussed.

## 2. Developing of CFD Model

**Figure 1:**
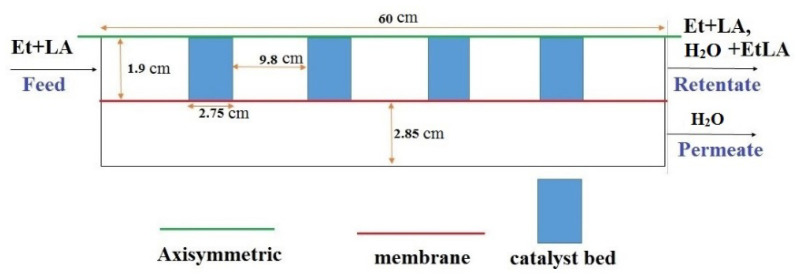
Scheme of the simulated MB-PVMR during LA-ESR reaction.

A CFD-based model was developed to simulate the performance of an MB-PVMR housing a poly(vinyl alcohol) (PVA) membrane, considering a two-dimensional and axisymmetric geometry. Figure 1 displays a simple scheme of the MB-PVMR (including four catalytic beds) adopted for EtLA production during LA-ESR reaction over a Smopex-101 catalyst. The main assumptions performed in the CFD simulations are summarized below:

- Isothermal condition;
- Steady-state regime;
- The film transport resistance at the interface of feed/membrane was considered negligible;
- Absence of concentration polarization effects;
- Membrane and catalyst not affected by deactivation.

### 2.1. Governing Physical Equations

The governing equations in nonporous and porous zones are given in Table 1. The source term S_i_ in the porous momentum equation is expressed as [25]:

(2) $$S=-\frac{\mu }{K_{\mathrm{br}}}\;u+\beta_{F}\left|u\right|u$$

where μ is the liquid mixture viscosity, K_br_ is permeability, and $\beta_{F}$ is the Forchheimer coefficient for packed-bed particles. K_br_ and $\beta_{F}$ are defined as:

(3) $$K_{\mathrm{br}}=\frac{d_{\mathrm{cat}}^{2}{\;\varepsilon }^{3}}{180{\left(1-\varepsilon \right)}^{2}}$$

(4) $$\beta_{F}=\frac{1\cdot 75\rho }{\sqrt{150K_{\mathrm{br}}\varepsilon^{3}}}$$

The effective diffusion in porous media,$\;D_{\mathrm{ei}},$ depends on the structure of the porous material and the phases involved. In saturated or partially saturated porous media, the effective diffusivity is defined as:

(5) $$D_{\mathrm{ei}}=\frac{\varepsilon }{\tau }D_{\mathrm{Li}}$$

The fluid tortuosity for the Millington and Quirk model is:

(6) $$\tau =\varepsilon^{-1/3}$$

where ε, ρ, d_cat_, and $D_{\mathrm{Li}}$ are the porosity, density of fluid, diameter of catalyst particle, and single-phase diffusion coefficients for the species in fluid, respectively. The porosity and particle diameter were set at 0.3124 and 0.01 mm, respectively. Porosity for the nonporous zone was set at 1. The flow pattern at the nonporous zone momentum equation is laminar flow. S_i_ in the porous species equation is the sink/source term of component i, which accounts for the addition or removal of component i into the system for permeation through the membrane. In this work, since only water permeates from the retentate to the permeate side, it appears as a sink term on the retentate side and a source term on the permeate side. In other words, S_i_ = 0 is considered for all components except for H_2_O, which is calculated as:

(7) $$S_{i}=\frac{{A\;J}_{H2O}M_{H2O}}{V}$$

where A is the membrane surface, *V* is the computational cell volume, M_H_2_O_ is the water molar weight, and J_H_2_O_ is the water permeating flux across the PVA membrane, calculated by Equation (8):

J_H_2_O_ = J_0,H_2_O_(_CH_2_O,permeate_ − _CH_2_O,retentate_) (8)

The water permeating flux (J_0,H_2_O_) is reported at different temperatures in Table 2 [25].

### 2.2. Chemical Kinetic Reactions

The empirical reaction rate of LA-ESR reaction, conducted by Russo et al. [26,27] using Smopex-101 heterogeneous catalyst and the report self-catalyzed of this reaction is used as follows:

(9) $$R_{1}=k_{1.0}c_{\mathrm{LA}}\left(c_{\mathrm{LA}}c_{\mathrm{Et}}-\frac{1}{K}c_{\mathrm{EtLA}}c_{w}\right)\left(\mathrm{self}-\mathrm{catalyzed}\right)\;$$

(10) $$R_{2}=k_{2.0}\rho_{\mathrm{cat}}\left(c_{\mathrm{LA}}c_{\mathrm{Et}}-\frac{1}{K}c_{\mathrm{EtLA}}c_{w}\right)\left(\mathrm{heterogeneous}\;\mathrm{catalyst}\;=\;\mathrm{Smopex}-101\right)$$

R_t_ = R_1_ + R_2_ (11)

$$r_{\mathrm{LA}}=r_{\mathrm{Et}}=-{\mathrm{Rt}\;\mathrm{and}\;r}_{\mathrm{LAEt}}=r_{H2O}=\mathrm{Rt}$$

where R_t_ and R_1_ are the reaction rate at the catalytic and non-catalytic beds, respectively. Moreover, rate constants are defined according to Equation (12).

(12) $$k_{i}=k_{i0}\;\exp\left(-\frac{E_{i}}{R}\left(\frac{1}{T}-\frac{1}{T_{\mathrm{ref}}}\right)\right)$$

In Equation (10), $\rho_{\mathrm{cat}}$ is the catalyst density and K the equilibrium constant expressed as:

(13) $$K=K_{\mathrm{ref}}\;\exp\left(-\frac{∆H}{R}\left(\frac{1}{T}-\frac{1}{T_{\mathrm{ref}}}\right)\right)$$

The parameters used of reaction rate constants are reported in Table 3.

### 2.3. Chemical-Physical Properties

The dependence of the liquid density on the temperature for each single component was evaluated according to the empirical expression reported below [27]:

(14) $$\rho_{i}={\mathrm{MW}}_{i}\cdot \frac{A_{i}}{B_{i}^{\left[1+{\left(1-\frac{T}{C_{i}}\right)}^{D_{i}}\right]}}$$

The coefficients of Equation (14) for each component are reported in Table 4.

The evaluation of the diffusion coefficient (D_i_) of the various components was carried out in accordance with the Wilke and Chang equation for liquid systems [28]:

(15) $$D_{i}=\frac{7\cdot 4*10^{-8}{\left({\varphi M}_{i}\right)}^{1/2}T}{\mu_{\mathrm{mix}}V_{i}^{0.6}}$$

where V_i_ represents the molar volume at the normal boiling point (cm^3^/mol), calculated through the inverse of Equation (14), and µ_mix_ is the viscosity of the reaction mixture (cP). The term ϕ is defined as association factor and depends on the nature of the chemical component (ϕ_LA_ = ϕ_EtLA_ = 1.0, ϕ_Et_ = 1.5 and ϕ_H_2_O_ = 2.6). The ϕM_i_ was therefore obtained for each component according to the Equation (16):

(16) $$\phi M_{i}=\overset{n-1}{\underset{j}{\sum }}x_{i}^{j}\cdot \phi_{i}\cdot {\mathrm{MW}}_{i}\;\left(n=4;j\neq i\right)$$

where $x_{i}^{j}$ is a molar fraction, in each step, determined for three components, using the i-th component and varying the j factor [28]. The temperature dependence on the viscosity was hence evaluated for each single component present in the reaction system, based on the empirical expression retrieved from the CHEMCAD database [29].

(17) $$\mu_{i}=\exp\left(A_{\mu i}+\frac{B_{\mu i}}{T}+C_{\mu i}\cdot \ln\left(T\right)+D_{\mu i}T^{E_{\mu i}}\right)$$

The coefficients of the Equation (17) for each component are reported in Table 5.

### 2.4. Boundary Conditions and Post Processing Definitions

In Table 6, the boundary conditions for retentate and permeate sides are shown. The following correlations were defined for describing the MB-PVMR performance in LA-ESR reaction:

(18) $$\mathrm{LA}-\mathrm{conversion}\;\left(\%\right)=\frac{{\mathrm{LA}}_{\mathrm{in}}-{\mathrm{LA}}_{\mathrm{out}}}{{\mathrm{LA}}_{\mathrm{in}}}*100\;$$

(19) $$\mathrm{Water}\;\mathrm{removal}\;\left(\%\right)=\frac{{H_{2}O}_{\mathrm{Permeate}}}{{H_{2}O}_{\mathrm{Permeate}}+{H_{2}O}_{\mathrm{retentate}}}*100$$

where LA_in_ and LA_out_ are the inlet and outlet levulinic acid molar flow rates, respectively, and H_2_O_retentate_ and H_2_O_permeate_ are the water molar flow rates in the retentate and permeate flow, respectively.

### 2.5. Numerical Method

Numerical simulations were performed using the commercial CFD package COMSOL Multiphysics 5.4, and the finite element method was used to solve the governing equations in the two-dimensional CFD model. Moreover, the pressure-velocity correction was performed using a coupling algorithm. Meanwhile, the equation solution was considered to be achieved when the residuals converged to values less than the magnitude of 10^−5^, and all the variable values were not changed with the iteration.

### 2.6. Mesh Independency

**Figure 2:**
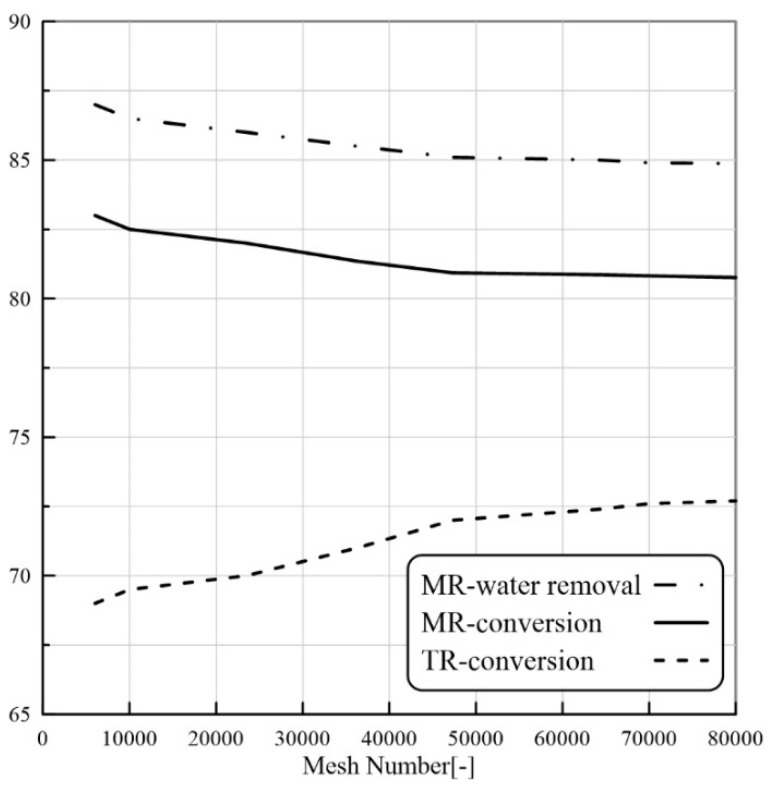
Effect of mesh number on the calculated LA conversion and water removal by CFD model (ET/LA:1.25, feed flow rate 7 mm^3^/s and T = 343 K, catalyst loading = 8.6 g).

Another objective of the preliminary CFD simulations was to carry out a grid independence test. For this purpose, CFD simulations were carried out using different grid densities to find out the values beyond which the results become grid-independent. The investigated mesh numbers were 6020, 23,478, 36,240, 64,256, and 80,000. These simulations were carried out for MBPMR configuration during LA-ESR reaction (reaction temperature 343 K, feed flow rate 7 mm^3^/s, and molar ratio of Et/LA:1.25 and W = 8.6 g). The results for the grid independence tests are shown in Figure 2. The tracked parameter is the LA conversion. The results depict that beyond 48,000 meshes, the conversion shows a slowly decreasing trend, whereas it becomes constant at higher grid numbers. The finest grid was, therefore, identified as the grid density at which the solution becomes grid-independent. This grid number (48,000 meshes) was considered in all the final simulations discussed in the subsequent sections.

## 3. Result and Discussion

### 3.1. Model Validation

**Figure 3:**
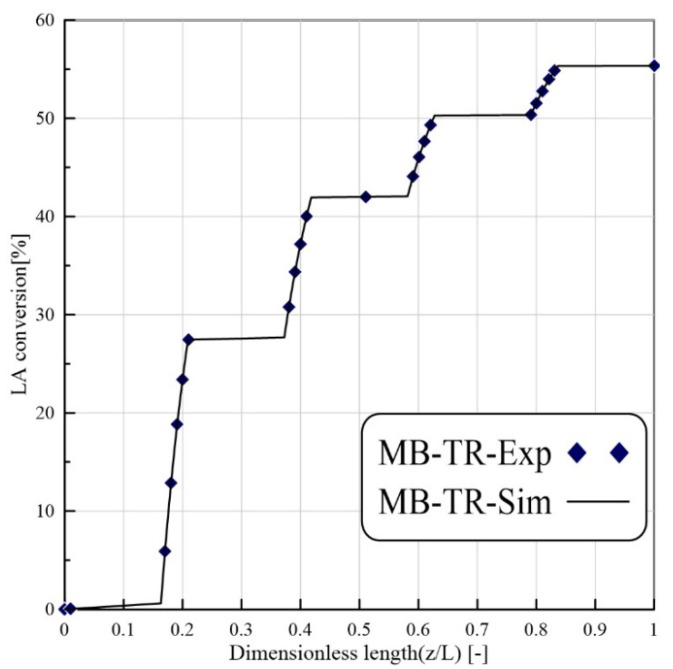
LA conversion versus reactor length for the MB-TR (feed flow rate: 16.7 (mm^3^/s), T = 333 K, Et/LA = 1:1, catalyst loading = 8.6 g) [27].

The exactness of the CFD model was confirmed using trial information of Russo et al. [27]. Figure 3 illustrates the LA conversion versus the reactor length for the MB-TR during the levulinic acid esterification reaction. The working conditions for the TR model are T = 333 K, Et/LA = 1:1 and volume flow rate = 16.7 mm^3^/s, W = 8.6 g. As shown in the latter figure, the simulated and the experimental values are in suitable agreement.

### 3.2. Concentration and Velocity Distributions

**Figure 4:**
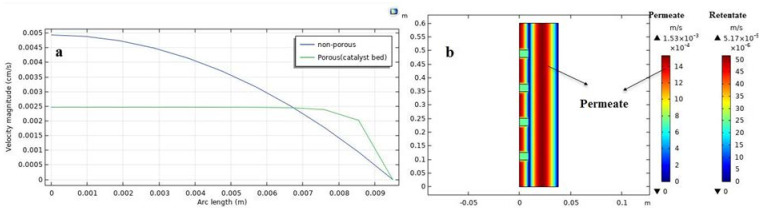
Velocity contour (m/s) during LA-ESR reaction in the MB-PVMR: (**a**) graph of velocity distribution; (**b**) contour of velocity achieved by CFD simulation (at feed molar ration ET/LA = 1.25, feed flow rate = 7 mm^3^/s, T = 343 K and catalyst loading = 8.6 g).

**Figure 5:**
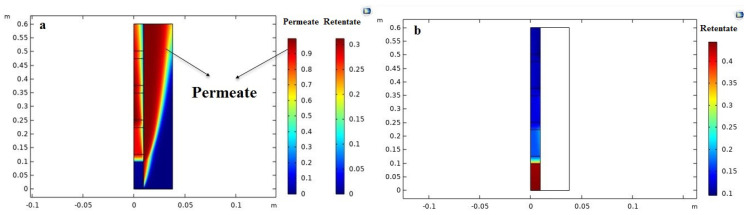
Mole fraction contours achieved by CFD simulation during LA-ESR reaction in MB-PVMR: (**a**) H_2_O mole fraction distribution; (**b**) LA mole fraction distribution simulation (at feed molar ration ET/LA = 1.25, feed flow rate = 7 mm^3^/s, T = 343 K and catalyst loading = 8.6 g).

The velocity distributions in the MB-PVMR, including the retentate and permeate zones, are shown in Figure 4b. The velocity values are indicated by the different colors of contours in agreement with the scale shown on the right side of the chart, where velocity distribution at the nonporous zone follows a parabolic form in both sides of the MB-PVMR. Indeed, the maximum values of velocity are observed in the central area of the permeate and retentate sides. On the other hand, in the porous zone (catalyst bed), the velocity distribution is uniform in the catalyst bed. Figure 4a illustrated the velocity profile in porous and nonporous zones. Mole fraction distribution of H_2_O in both sides of the MB-PVMR is the most important parameter for the optimization of the process. Figure 5 sketches the distribution of H_2_O and levulinic acid concentrations as well as the permeation of water by membrane during production. The mass transfer flux in the shell and tube sides is composed of diffusion and convection. The convection mechanism tends to transfer H_2_O to the outlet of MB-PVMR owing to the high contribution of velocity in the vertical direction. The H_2_O is transferred toward the membrane by diffusional mass transfer that is responsible for the H_2_O removal.

### 3.3. Evaluation of Operating Parameters Effects

As reported in Table 7, after the preliminary CFD analysis, simulations were carried out to understand the effects of the most important operating parameters on the performance of the dense MB-PVMR in terms of LA conversion and water removal during LA-ESR reaction. The operating parameters were firstly checked in the model validation and, successively, we simulated the membrane reactor analyzing the mutual influence of them each other. This approach made it possible to understand from a theoretical point of view which kind of combination of operating conditions may determine better performance in terms of simulated conversion and water removal. In particular, the simulation sets can be subdivided into four parts, in which reaction temperature, catalyst loading, feed molar ratio, and feed flow rate values were changed for four cases of MB-PVMR and MB-TR.

#### 3.3.1. Effect of Feed Molar Ratio on PVMR Performance

**Figure 6:**
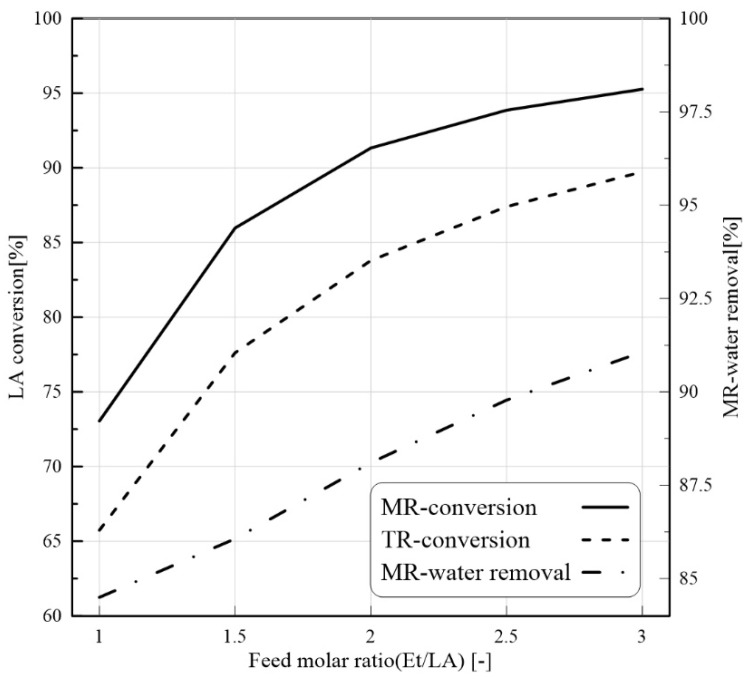
LA conversion for MB-PVMR and MB-TR and water removal for MB-PVMR versus feed molar ratio at feed flow rate = 7 mm^3^/s, T = 343 K, and catalyst loading = 8.6 g.

A further parameter that strongly affects the MB-PVMR performance is the feed molar ratio (Et/LA). High conversion of LA in higher content of Et is reachable. As shown in Figure 6, by increasing the feed molar ratio, LA conversion is enhanced. According to the LA-ESR reaction, an increase in Et/LA ratio may shift the reaction toward the products. Therefore, according to Le Chatelier’s principle, the LA conversion and water production can be improved at a higher Et/LA value. In the meantime, H_2_O removal is also improved (Figure 6). By considering the H_2_O compositions in both permeate and retentate sides, the H_2_O permeation driving force is increased at a higher feed molar ratio.

#### 3.3.2. Effect of Feed Flow Rate on PVMR Performance

**Figure 7:**
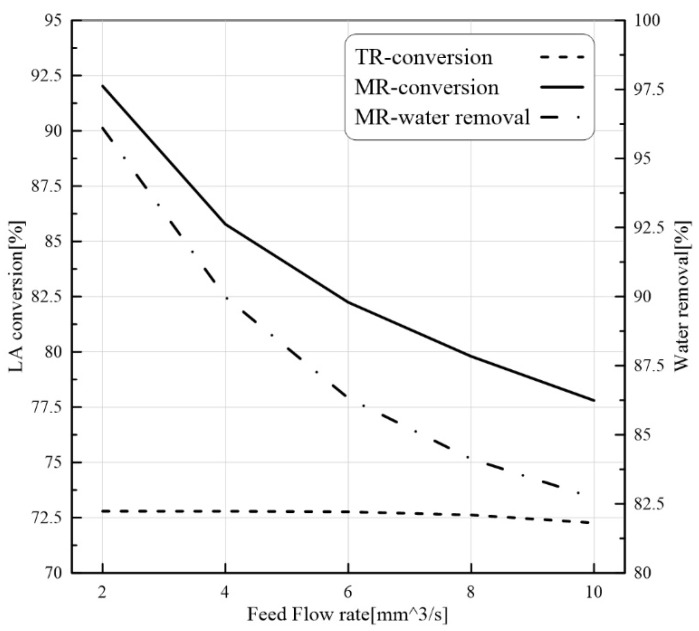
LA conversion for MB-PVMR and MB-TR and water removal for MB-PVMR versus feed flow rate at ET/LA feed molar ratio = 1.25, T = 343 K, and catalyst loading = 8.6 g.

Residence time can be directly responsible for the conversion variation. It is clear that a higher flow rate may be responsible for a reduction in the residence time. As illustrated in Figure 7, an increase in feed flow rate reduced LA conversion. Similar to the previous case, LA conversion in the MB-PVMR is higher than that of the MB-TR. The higher the amount of H_2_O in the reaction zone, the higher the membrane surface required to overcome the reduction in H_2_O removal. Therefore, under a constant membrane area, the H_2_O removal is depleted. So, a feed flow rate increase from 2 to 10 mm^3^/s reduced the LA conversion from ~ 92% to ~ 78% in the MB-PVMR and from ~ 72.8% to 72.26% in the MB-TR.

#### 3.3.3. Effect of Catalyst Loading on PVMR Performance

**Figure 8:**
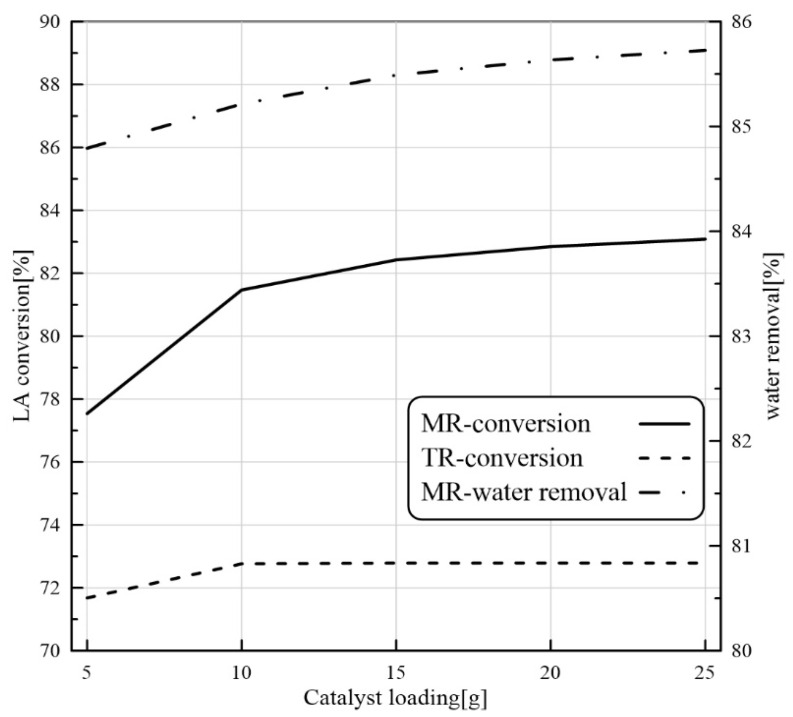
LA conversion for MB-PVMR and MB-TR and water removal for MB-PVMR versus catalyst loading at feed molar ratio = 1.25, T = 343 K, and feed flow rate = 7 mm^3^/s.

The catalyst performance during the reaction process is restricted by the catalyst load. Figure 8 depicts the impact of the catalyst load on the LA conversion and H_2_O removal in the MB-TR and MB-PVMR. Looking at the profiles of Figure 8, it is possible to observe that the increase in the catalyst load promotes the LA conversion. A slightly higher LA conversion in the MB-PVMR than that in the MB-TR is observed. The reason for that is attributed to the water separation in the MB-PVMR. In fact, by increasing the catalyst load, the conversion is enhanced and, therefore, H_2_O production is improved, leading to an increase in the driving force for a larger H_2_O removal.

#### 3.3.4. Effect of Temperature Reaction on PVMR Performance

**Figure 9:**
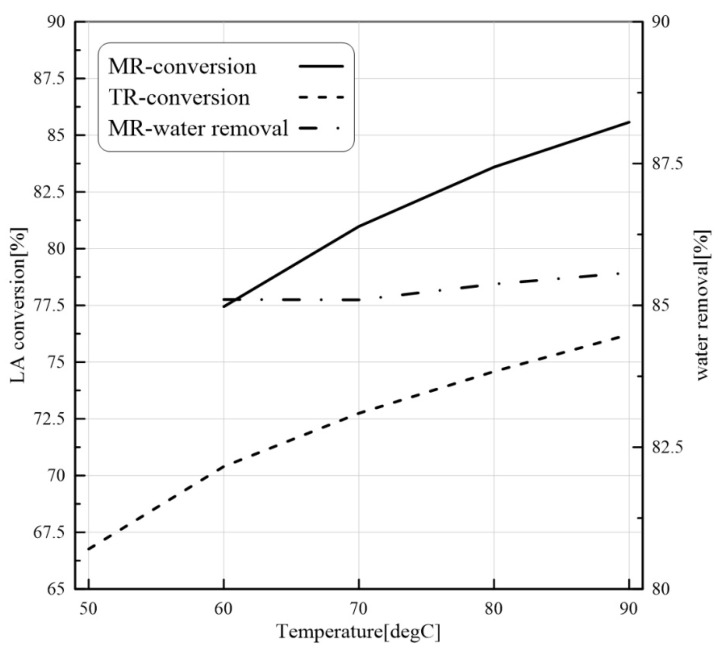
LA conversion for MB-PVMR and MB-TR and water removal for MB-PVMR versus T at ET/LA = 1.25, feed flow rate = 7 mm^3^/s, and catalyst loading = 8.6 g.

On the basis of reaction rate (Equation (11)) and H_2_O permeation flux (Table 1), which depends on the temperature through an Arrhenius-type equation, by raising the temperature, both LA conversion and H_2_O permeation flux increase. The prediction of CFD modeling in both MB-TR and MB-PMR as a function of the reaction temperature is shown in Figure 9.

LA conversion increases from 77.5% to 86% and H_2_O removal from 77.5% to 79% as the reaction temperature raises from 323 to 363 K. The increase in the H_2_O removal reveals that the effect of temperature on H_2_O permeation is more relevant than its effect on water production. On the other hand, due to a higher H_2_O removal at higher reaction temperatures, the difference between LA conversion in MB-PVMR and MB-TR results to be consequently larger.

## 4. Conclusions

In this theoretical work, LA-ESR reaction by PVMR use was studied for producing ethyl levulinate as a significant fuel additive. An isothermal and 2D CFD-based model was adopted to investigate the performance of multi-bed PVMR configuration in terms of LA conversion and H_2_O removal, comparing the results with an equivalent multi-bed TR. The simulation analysis was particularly focused on the effects of several parameters such as reaction temperature, feed molar ratio, feed flow rate, and catalyst loading on the reactors, and the simulations well matched the experimental literature data. The most important aspect of this work was that, under all the considered operating conditions, the performance of the MB-PVMR was always better than those of the equivalent MB-TR. Furthermore, the best simulation results were reached at 343 K, 2 bar, and feed molar ratio equal to 3, obtaining a levulinic acid conversion equal to 95.3% and 91.1% of water removal. As a further result, the CFD tool was capable of predicting the optimal conditions to operate the MB-PVMR in this work. It pointed out also the benefits and drawbacks of the MB-PVMR during ESR reaction, demonstrating how it may constitute a valid strategy for the scale-up of this technology and the future commercialization of PVMR systems in added-value ester production processes for developing fuel additive industries.

## Figures and Tables

**Table 1:** Governing equations at the CFD model.

Retentate	Permeate
Continuity equation: ∇·(ρ·u·ε)=0	Continuity equation: ∇·(ρ·u)=0
Momentum equation: $\frac{\rho }{\varepsilon^{2}}\left(\left(u\cdot \nabla \right)\frac{u}{\varepsilon }\right)=\nabla \cdot \left[-pI+\frac{\mu }{\varepsilon }-\frac{2\mu }{3\varepsilon }\left(\nabla u\right)I\right]+S$	Momentum equation: $\rho \left(u\cdot \nabla \right)u=\nabla \cdot \left[-p_{r}I+\mu \left(\nabla u+{\left(\nabla u\right)}^{\vartheta_{r}}\right)\right]$
Species equation: $u\cdot \nabla c_{i}=\nabla \cdot \left[\left(D_{ei}\right)\nabla c_{i}\right]+R_{i}+S_{i}$	Species equation: $\nabla \cdot \left(-D_{i}\nabla c_{i}+uc_{i}\right)=0$

**Table 2:** Value of water permeating flux for PVA membrane at different temperatures [25].

Temperature (°C)	J_0,H_2_O_ (L/m^2^·h)
60	3.50
70	3.52
80	3.87
90	4.2

**Table 3:** Value of rate constant parameters used in the simulations of LA-ESR reaction [26,27].

Parameter	Name	Value	Units
∆H	Enthalpy	15.14	kJ/mol
E_1_	Activation energy	32.51	kJ/mol
E_2_	Activation energy	39.38	kJ/mol
K_ref_	Equilibrium const.	3.18	-
k_1,0_	Rate const.	5.74 ×10^−14^	(m^3^/mol)^2^/s
k_2,0_	Rate const.	5.32 ×10^−10^	(m^3^/mol)⋅(m^3^/kg)/s
T_ref_	Temperature ref.	333	K

**Table 4:** Numerical values of the coefficients used to calculate the density of each component.

**Coefficients**	**LA**	**Et**	**EtLA**	**H_2_O**
A_i_	0.754	1.65	0.528	5.46
B_i_	0.258	0.276	0.246	0.305
C_i_	738	514	666	647
D_i_	0.220	0.233	0.286	0.081

**Table 5:** Coefficients used to calculate the viscosity of each component as a function of T.

**Coefficients**	**LA**	**Et**	**EtLA**	**H_2_O**
A_μi_	−12.873	7.874	−1.3913	−51.964
B_μi_	2295.7	781.98	1034.8	3670.6
C_μi_	−0.043631	−3.0418	−1.4837	5.7331
D_μi_	0	0	0	−5.3495 × 10^−29^
E_μi_	0	0	0	10

**Table 6:** Boundary conditions at the retentate and permeate sides.

Position	Retentate Side	Permeate Side
Z = 0	Inflow	-
Z = L	Outflow	Outflow
R = red line	Water flux	Water flux
R = green line	$\frac{\partial c}{\partial r}=0$	$\frac{\partial c}{\partial r}=0$

**Table 7:** The investigated operating conditions for the MB-PVMR and MB-TR during LA-ESR reaction.

**Operating Parameters**	**Temperature Effect**	**Feed Flow Rate Effect**	**Et/LA**	**Catalyst Loading**
T (K)	various	343	343	343
Feed flow rate (mm^3^/s)	7	various	7	7
Et/LA	1.25	1.25	various	1.25
Catalyst loading (g)	8.6	8.6	8.6	various

## Abbreviations and Nomenclature

A	Membrane surface (m)
CFD	Computational fluid dynamic
C_i_	Concentration of component i (mol/m^3^)
C_H_2_O,permeate_	Water concentration in the permeate side
C_H_2_O,retentate_	Water concentration in the permeate side
d_cat_	Diameter of catalyst particle (m)
$D_{\mathrm{Li}}$	Single-phase diffusion coefficients (m^2^/s)
$D_{\mathrm{ei}}$	The effective diffusion in porous media(m^2^/s)
E_1_	Activation energy (kJ/mol)
E_2_	Activation energy (kJ/mol)
Et	Ethanol
EtLA	Ethyl levulinate
J_H_2_O_	Water permeating flux (mol/m^2^/s)
J_0,H_2_O_	Water permeability
K	Equilibrium constant (-)
k_1,0_	Rate constant ((m^3^/mol)^2^/s)
k_2,0_	Rate constant ((m^3^/mol)⋅(m^3^/kg)/s)
K_br_	Permeability (m^2^)
K_ref_	Equilibrium cst (-)
LA	Levulinic acid
LA-ESR	Livulinic acid esterification
${\mathrm{MW}}_{i}$	Molecular weight of component i (mol/g)
NOx	Nitrogen oxides
PV	Pervaporation
PVA	Poly(vinyl alcohol)
PVMR	Pervaporation membrane reactor
r_i_	Reaction rate of component i (mol/m^3^/s)
S_i_	Sink/source term of component i (kg/m^3^/s)
SO_x_	Sulfur oxides
T_ref_	Temperature ref (K)
u	Velocity (m/s)
V	Computational cell volume (m^3^)
V_i_	Molar volume at normal boiling point (cm^3^/mol)
W	Weight of catalyst (g)
$\beta_{F}$	Forchheimer coefficient
∆H	Enthalpy (kJ/mol)
ε	Porosity (-)
μ	Viscosity (cP)
µ_mix_	Viscosity reaction mixture (cP)
ρ	Density (kg/m^3^)
$\rho_{\mathrm{cat}}$	Catalyst density (kg/m^3^)
τ	Tortuosity
ϕ	Association factor (-)
